# Relative Contribution of Metabolic Syndrome Components in Relation to Obesity and Insulin Resistance in Postmenopausal Osteoporosis

**DOI:** 10.3390/jcm13092529

**Published:** 2024-04-25

**Authors:** Daniela Greere, Florin Grigorescu, Dana Manda, Gabriela Voicu, Corinne Lautier, Ileana Nitu, Catalina Poiana

**Affiliations:** 1 Department of Endocrinology, Faculty of General Medicine, Carol Davila University of Medicine and Pharmacy, 8 Eroii Sanitari Bd., 050474 Bucharest, Romania; endoparhon@gmail.com; 2 Department of Clinical Endocrinology, C. I. Parhon Institute of Endocrinology, 34-38 Aviatorilor Bd., 011863 Bucharest, Romania; 3 Institut Convergences Migrations, Collège de France, 1440 Ave des Orchidées, 34980 Saint Clément de Rivière, France; florin.grigorescu@inserm.fr; 4 Molecular Cellular and Structural Endocrinology Laboratory, C. I. Parhon Institute of Endocrinology, 34-38 Aviatorilor Bd., 011863 Bucharest, Romania; dana.manda@gmail.com; 5 Nuclear Medicine Laboratory, C. I. Parhon Institute of Endocrinology, 34-38 Aviatorilor Bd., 011863 Bucharest, Romania; voicugabi@gmail.com; 6 Qualisud, Univ Montpellier, Avignon Université, Centre de Coopération Internationale en Recherche Agronomique Pour le Développement, Institut Agro, Institut de Recherche Pour le Développement, Université de La Réunion, 15 Ave Charles Flahault, 97400 Montpellier, France; corinne.lautier@umontpellier.fr; 7 Department of Cardiology, C. I. Parhon Institute of Endocrinology, 34-38 Aviatorilor Bd., 011863 Bucharest, Romania; ileananitu2003@yahoo.com

**Keywords:** osteoporosis, insulin resistance, metabolic syndrome, HOMA index

## Abstract

**Introduction**. Osteoporosis (OP) affects 30% of postmenopausal women, often complicated by metabolic syndrome (MetS) with a still controversial role. We aimed to characterize MetS and its components in relation to bone mineral density (BMD), body mass index (BMI), and insulin resistance. **Methods**. Patients (*n* = 188) underwent DEXA scans, spine X-rays, and metabolic and hormonal investigations, including bone biomarkers, muscular strength, and physical performance tests, while insulin resistance was evaluated by the Homeostasis Model Assessment (HOMA-IR). **Results**. Patients with a normal BMD or osteopenia (*n* = 68) and with OP (*n* = 120) displayed 51.5% and 30.8% of MetS, but without differences in insulin resistance. When BMD was studied as a function of the cumulative MetS criteria and centiles of BMI, lower levels of BMD were observed beyond an inflection point of 27.2 kg/m^2^ for BMI, allowing for further stratification as lean and overweight/obese (OW/OB) subjects. In contrast with lean individuals (*n* = 74), in OW/OB patients (*n* = 46), MetS was associated with HbA1c (*p* < 0.0037, OR 9.6, 95% CI [1.64–55.6]) and insulin resistance (*p* < 0.0076, OR 6.7, 95% CI [1.49–30.8]) in the context where BMD values were lower than those predicted from BMI in non-OP subjects. In OP patients with fragility fractures (31% of MetS), glycemia also appeared to be the dominant factor for MetS (*p* < 0.0005, OR 4.1, 95% CI [1.63–10.39]). **Conclusions**. These data indicate a detrimental effect of insulin resistance in MetS on OP patients, while the prevalence of the syndrome depends on the proportion of obesity. These findings provide new insights into the pathogenic role of MetS and reveal the need to consider different strata of BMI and insulin resistance when studying postmenopausal OP.

## 1. Introduction

Osteoporosis (OP) is a multifactorial disease characterized by reduced bone strength resulting from a diminished bone mass and altered bone architecture, predisposing individuals to fractures [1,2]. Given the increased susceptibility to fractures and the associated morbidity and mortality, OP emerges as a pressing public health concern, particularly among the older population. According to the International Osteoporosis Foundation, the estimated global OP prevalence stands at 18.3%, signifying that approximately one in three women aged 50 or older will experience osteoporotic fractures during their lifetime [3]. In central Europe, in countries such as Romania, OP’s prevalence among women has been reported at 20.5% [4].

In the aging population, one significant concern revolves around the progressive deterioration of metabolic health, encompassing the emergence of obesity and its associated complications, such as Type 2 diabetes mellitus (T2D) and metabolic syndrome (MetS), both hallmarked by insulin resistance [5]. MetS, also known as “syndrome X” or the “deadly quartet”, is defined by a constellation of metabolic and cardiovascular risk factors, including obesity, hyperglycemia, high blood pressure (HBP), and abnormal lipid profiles [6,7]. This intricate syndrome becomes more prevalent with an advancing age and has garnered specific attention in postmenopausal women [8]. Its relationship with systemic or bone-specific insulin resistance remains, however, complex, as recently reviewed by us [9]. Although systemic insulin resistance fundamentally characterizes MetS, this feature has not been included as a criterion for the definition of MetS in clinical studies.

Prior investigations into the relationship between MetS and OP in postmenopausal women primarily focused on the prevalence of MetS, yielding conflicting results. Some studies suggest an elevated prevalence, interpreted as possibly predisposing to OP, while others have reported a lower prevalence, implying an apparent protective effect. This discrepancy has spurred extensive reviews in the literature, all of which have reached the consensus that the association between MetS and OP remains enigmatic [10,11,12]. Intuitively, MetS might be considered to exacerbate comorbidities in older people due to its associated cardiovascular complications, making the observed lower or unchanged prevalence of MetS in postmenopausal OP somewhat unexpected.

While several factors may contribute to the disparities among studies, such as variations in MetS definitions, ethnicity, and lifestyle, one pivotal concern lies in quantifying insulin resistance and modalities to study the contribution of MetS components in relation to obesity. The Homeostasis Model Assessment (HOMA-IR) stands as a simple and robust parameter for assessing insulin resistance in epidemiological studies [13]. However, only a limited number of studies of MetS have measured insulin resistance within the OP population. In the general population, it has been firmly established that HOMA-IR positively correlates with the cumulative MetS criteria, which can offer valuable insights into the role of MetS components [14,15], but the relation with bone mineral density (BMD) is poorly studied. Another layer of complexity pertains to the relative influence of obesity [16,17]. Obese women, through mechanisms such as the mechanical loading of bone or hormonal alterations (e.g., estrogen synthesis by adipose tissue or elevated insulin levels), often exhibit a higher BMD, suggesting a protective role [17,18,19]. The protective effect of weight gain in post-menopausal women is an old assumption. However, more recent studies have indicated that obesity, despite being associated with an increase in BMD in OP, does not protect against fragility fractures [16]. Moreover, this protective effect becomes challenging to demonstrate in OP, especially among older patients who frequently present a leaner phenotype [19]. A recent study in the Taiwanese population indicated that, in the context of multifactoriality, MetS could increase the risk for more severe low bone density, but this effect is modulated by the degree of obesity, namely the body mass index (BMI) [20]. The study of MetS in OP (especially in the setting of fractures) is additionally complicated by the association of reduced muscle mass and strength in patients with altered bone health, a condition referred to as osteosarcopenia [21,22].

In light of all these complexities, this study aimed to characterize MetS and the relative contribution of its various components in postmenopausal OP through a thorough analysis of the correlation between BMD, the cumulative MetS criteria, measured insulin resistance, and degree of obesity. In this paper, we describe a biphasic variation of BMD as a function of BMI, allowing for a novel stratification of patients as lean and overweight/obese (OW/OB) beyond an inflection point of BMD, revealing the role of insulin resistance.

## 2. Materials and Methods

### 2.1. Population and Ethical Statement

This cross-sectional, prospective study included women recruited at the C.I. Parhon National Institute of Endocrinology, Bucharest, Romania, during the period from 28 May 2020 to 1 April 2022. The inclusion criteria were as follows: (1) women aged 50–75 years; (2) time from menopause ≥1 year; and (3) Caucasian (Romanian) origin. No limit was considered for BMI. Treatment for OP or for MetS was not considered as an exclusion criterion. Excluded were all forms of secondary osteoporosis after hormonal examination, as well as severe chronic diseases (except for T2D), such as rheumatoid arthritis, kidney disease, or inflammatory bowel diseases. The Institutional Ethical Committee approved the research protocol, and signed informed consent was obtained from each patient in accordance with the Helsinki Declaration [23].

### 2.2. Clinical and Biochemical Assessments

Body weight, height, and waist circumference (WC) were recorded, and BMI was calculated as weight in kilograms (kg) divided by the square of height in meters. Before any type of stratification, the cutoffs of 25 and 30 kg/m^2^ were used to define overweight (OW) and obesity (OB), respectively.

Venous blood samples were collected after an overnight fast. A comprehensive set of blood tests was performed using a clinical chemistry autoanalyzer (Cobas c 501, Roche Diagnostics), including blood count, fasting glucose, urea, creatinine, uric acid, albumin, total protein, total cholesterol, high-density lipoprotein cholesterol (HDL-C), low-density lipoprotein cholesterol (LDL-C), triglycerides (TG), total and ionized calcium, magnesium, phosphate, alkaline phosphatase (ALP), erythrocyte sedimentation rate (ESR), C-reactive protein (CRP), and fibrinogen. Insulin, (25-OH) vitamin D, parathyroid hormone (PTH), osteocalcin, procollagen type I N-terminal propeptide (P1NP), and beta-Crosslaps were measured using electrochemiluminescent immunoassay (ECLIA) with a Cobas e 601 module (Roche Diagnostics).

MetS diagnosis was based on the presence of at least 3 of the harmonized criteria of the National Cholesterol Education Program (NCEP) and Adult Treatment Panel-III (ATP-III), which include: (1) abdominal obesity based on a WC of ≥88 cm, (2) a high TG level of ≥1.7 mmol/L, (3) low HDL-C of <1.03 mmol/L, (4) HBP with a systolic blood pressure (SBP) of ≥130 mmHg and/or diastolic blood pressure (DBP) of ≥85 mmHg, and (5) high fasting glucose levels of ≥5.36 mmol/L, or current treatment with antihyperlipidemic, antihypertensive, or hypoglycemic agents, respectively [24]. Insulin resistance was assessed using HOMA-IR or as a nominative variable defined as having HOMA-IR values above the cutoff of 1.92, which was calculated from the fasting insulin levels of lean patients without OP + 2 SEM, as previously described [25,26].

### 2.3. Skeletal Assessment

All participants underwent a dual X-ray absorptiometry analysis (DEXA) using GE-Lunar Prodigy Pro (GE Healthcare, Machelen, Belgium). The study included women with a normal BMD, newly diagnosed or already treated osteopenia, and primary OP. For postmenopausal women, a normal BMD was defined as a T score between +1 and −1 SD, while osteopenia was defined as a T score between −1 and −2.5 SD. OP was defined according to the American Association of Clinical Endocrinologists (AACE) and National Osteoporosis Foundation guidelines [27]. We used specific AACE criteria in postmenopausal women based on any of the following: (1) a T-score of −2.5 or below in the lumbar spine, femoral neck, total proximal femur, or 1/3 radius; (2) a low-trauma spine or hip fracture, regardless of BMD; (3) a T-score between −1.0 and −2.5 and fragility fractures of the proximal humerus, pelvis, or distal forearm; and (4) a T-score between −1.0 and −2.5 and a high FRAX^®^ fracture probability based on country-specific thresholds [28]. The FRAX PLUS (TBS) score for the evaluation of 10-year risk for low-energy fractures was computed on the country-specific website (https://www.fraxplus.org/). In addition, severe OP was diagnosed based on the WHO criteria, namely the association of a T score equal to or less than −2.5 SD (e.g., <−3) and fragility fractures [29]. Patients with osteopenia and OP underwent lateral spine radiography, and the additional prevalence of fragility fractures was established from their medical history. The bone microarchitecture was evaluated by the Trabecular Bone Score (TBS), calculated for the lumbar spine at L1–L4.

### 2.4. Muscular Strength and Physical Performance

Muscular strength and physical performance were evaluated using handgrip strength (HGS), the chair stand test (CST), gait speed, the timed up and go test (TUG), and the Tinetti assessment tool, as described in Table S1 [30].

### 2.5. Statistical Analysis

The study was powered using the Raosoft program v4.5 (www.Raosoft.com), considering a prevalence of 30% of MetS in the general population and imposing a population size of at least *n* = 112 for a 90% confidence level. In the inter-group analysis, numerical variables were tested by the non-parametric Mann–Whitney test, while for multivariate ANOVA, the interaction factor α was set at 5%. Nominal variables were analyzed using the χ^2^ test. The relative contribution of components of MetS were studied in logistic regression and appreciated by statistical significance and values of OD. Logistic regression for MetS was performed using the descent method to obtain the *p* values, odds ratio (OR), and 95% confidence interval (CI), as previously described [15]. Significance was considered at *p* < 0.05.

Statistical analysis was performed in two main steps. In the first step, we analyzed the contribution of various components for MetS in unstratified groups as a baseline for our study. The patients were classified according to AACE as having a normal BMD or osteopenia (defined herein as non-OP individuals) and osteoporosis (OP). In a second step, a new analysis based on several original observations was performed in the population after stratification as a function of BMI. The first new observation concerned the correlation of BMD and HOMA-IR with the cumulative criteria for MetS (categories of none to 1, 2, and ≥3, criteria) considering (ANOVA) either the whole population or in each group. As a standard, we considered the BMD at the femoral neck, but all skeletal sites were tested. The results are expressed as genuine values of BMD, but we also tested values adjusted for BMI and for age. The significance in ANOVA among the categories of the cumulative criteria of MetS was determined using the post hoc Student–Newman–Keuls test. The second observation concerned the relation between BMD and the degree of obesity, considered as centiles of BMI (categories from A to F), with specific cutoff values of 21.0, 23.8, 27.2, 31.1, and 35.5 kg/m^2^, respectively. The inflection point of BMD was obtained by an inspection of the residuals of BMD, calculated by subtracting the predicted values of BMD (based on the BMD–BMI relationship in the non-OP population) from the genuine BMD values. Analysis was performed for each anatomical site. The inflection point with a cutoff value for BMI of 27.2 kg/m^2^ allowed for the stratification of OP patients as lean and overweight/obese (OW/OB) individuals. Other stratifications were also tested considering central obesity (COB) based on WC or lean versus obese (OB) patients based on the classical BMI cutoff of 30.0 kg/m^2^. To resume the evaluation of muscular strength and physical performance, we composed a statistical instrument (SUM^stat^) considering none, 1, 2, 3, 4, or all 5 muscular tests outside the normal values. This binary (0/1) SUM^stat^ parameter was displayed in tables and used in logistic regression. Furthermore, the values of each muscular test are indicated in the Supplementary Saterial. The statistical analysis was performed using the Statview 5.0 program, Abacus Concepts, Berkeley, CA, USA, as previously described [15,25].

## 3. Results

### 3.1. Group Analysis

The clinical and laboratory features of 188 patients, classified according to the AACE guidelines, are presented in Table 1.

Patients with OP were of a more advanced age, had a 2-fold lower prevalence of obesity based on BMI, and a 1.3-fold lower COB based on WC. Severe OP with fragility fractures represented 48.3% of OP cases. There was no significant difference in the prevalence of insulin resistance or HOMA-IR values. BMD was decreased at all anatomical sites, as was the TBS at L1–L4. Except for a slight decrease in P1NP levels (*p* < 0.02), other serum hormones and bone turnover markers remained non-significantly changed. Muscular strength and physical performance tests with abnormal values (detailed in Table S2) were 2-fold more frequent in OP patients. The population contained 42.0% patients currently treated for OP and 57.9% patients untreated in the last 1 year, composed of patients naive to treatment (44.1%) and patients in a drug holiday period (13.8%).

In OP patients, MetS was 1.7-fold less prevalent than in non-OP patients (*p* < 0.031), concordant with a 2.3-fold decrease in obesity and significantly lower prevalence rates of four ATP-III criteria (high TG appeared as a trend and low HDL was non-significant). The relative contribution of components of MetS was analyzed in logistic regression in each group by comparing patients with and without MetS (Table S3). In non-OP patients, influential components were BMI (*p* < 0.0001, OR 1.21, 95% CI [1.09–1.35]), while high TG appeared as a trend with *p* < 0.01, OR 5.23, 95% CI [0.9–29.9]. In OP patients, the same factors were determinant: BMI (*p* < 0.0014, OR 1.2, 95% CI [1.06–1.34]), high TG with *p* < 0.0002, OR 6.5, 95% CI [2.29–18.9], and, in addition, fasting glycemia (*p* < 0.004, OR 3.55, 95% CI [1.25–10.09]). HOMA-IR values, insulin resistance prevalence, and bone-related parameters were not significant, although actual values of BMD were increased in MetS (except for the radius in OP patients). These data may be explained by the heterogeneity of OP patients, encompassing both lean and obese subjects or combining simple OP and severe OP with fractures. To test these hypotheses, we examined the relationship between BMD and insulin resistance as a function of the cumulative criteria for MetS and various degrees of obesity.

### 3.2. Determinants of BMD

In the whole population, BMD (femoral neck) was explained by MetS (*p* < 0.0001, α = 0.99, ANOVA) with the confounding factors BMI, obesity, or WC, as well as glycemia, insulin levels, and HOMA-IR. Age explained BMD independently (*p* < 0.0001, α = 1.0), as did uric acid (*p* < 0.0035, α = 0.85), fibrinogen (*p* < 0.0102, α = 0.73), CRP (*p* < 0.0212, α = 0.602), and OCN (*p* < 0.026, α = 0.6). Obesity defined by a BMI of ≥ 30 kg/m² best explained the elevated BMD at all anatomical sites with an equivalent significance (*p* < 0.0001, α = 1.0). The same factors explained BMD when ANOVA was performed separately in each group (non-OP and OP patients), except for the additionally slightly significant P1NP in OP patients (*p* < 0.027, α = 0.61). To test BMD in logistic regression, we divided the BMD values into nominal categories (A and B) under and over the median value (0.768), respectively. Again, BMI remained the most influential factor with *p* < 0.0001, OR 1.21, 95% CI [1.12–1.310] in the entire population or in each group, with an additional effect as a trend for fibrinogen (*p* < 0.0042, OR 1.036, 95% CI [1.003–1.35]) in non-OP and for OCN (*p* < 0.0306, OR 0.94, 95% CI [0.90–1.00]) in the OP group. These data indicate that the components of MetS are very intricate, in which the degree of obesity plays the role of the driving force for BMD.

### 3.3. BMD as Function of Cumulative MetS Criteria and Obesity

**Figure 1:**
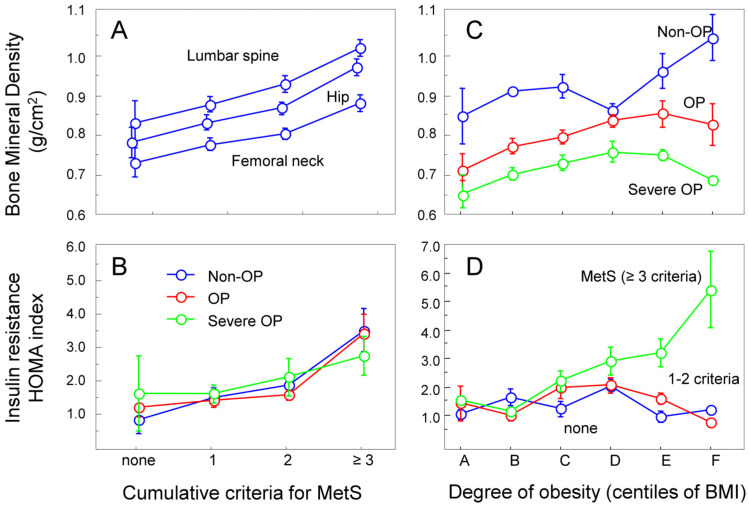
**Correlation between BMD and HOMA-IR index for insulin resistance as function of cumulative criteria for MetS and degree of obesity.** (**A**) BMD at femoral neck, hip, and lumbar spine as function of cumulative criteria of MetS. (**B**) Variation in HOMA-IR as function of cumulative criteria of MetS. (**C**) Variation in BMD at femoral neck as function of centiles of BMI in non-OP, OP, and severe OP with fractures. (**D**) Variation in HOMA-IR index in patients without any criterion for MetS, 1, 2, and 3 to 5 criteria for MetS.

A strong correlation was found between the cumulative MetS criteria and BMD across all anatomical sites (Figure 1A), including the radius, albeit at a lower level (Figure S1). Variance in BMD was significantly explained by the cumulative MetS criteria (*p* < 0.0001, α = 0.99, ANOVA). The most significant interaction was observed at the hip (α = 1), while at the radius, α was only 0.89. The post hoc Student–Newman–Keuls tests indicated the significance between category “none” versus 1, 2, and ≥3 MetS criteria. The significance disappeared when BMI was included as a co-factor (*p* < 0.51), clearly indicating the dependency on obesity. The correlation between BMD and BMI was also significant in Spearman test with *p* < 0.0001, r = 0.45 for the femoral neck and slightly different for the lumbar spine (r = 0.49), hip (r = 0.57), and radius (r = 0.50). A correlation was also observed with adjusted BMD values for age. A similar good correlation with the cumulative MetS criteria was found for the HOMA-IR index (*p* < 0.0001, α = 0.99), with no difference between individuals with or without OP or severe OP (Figure 1B). The Spearman test for HOMA-IR and BMI indicated *p* < 0.0001, r = 0.43, confirming the prevailing notion that obesity plays a significant role. Understanding the role of MetS components remains challenging because, on the one hand, an increase in the cumulative MetS criteria suggests more severe metabolic abnormalities (likely to occur in older patients with severe OP), and on the other hand, a higher BMI is associated with a higher BMD (likely to reduce the risk of OP).

To resolve this challenging problem, we further analyzed the correlation of BMD with the degree of obesity displayed as centiles of BMI values (Figure 1C). Notably, at lower BMI values (centiles categories A–C), a positive correlation persisted in all patient groups, but for higher BMI values (categories D–F), the BMD curves were blunted and decreased for OP, while those for non-OP patients continued to increase with higher BMI values. In this biphasic effect, the apparent inflection point was 27.2 kg/m^2^ for BMI, which was also visualized by plotting the negative residuals of BMD in OP from predicted values in non-OP patients (Figure S2). The decrease in BMD was more pronounced in severe OP, suggesting more severe abnormalities in the presence of insulin resistance. As shown in Figure 1D, high HOMA-IR values were compatible with the notion of insulin resistance when the BMI exceeded 23.8 kg/m^2^ in patients with three or more criteria for MetS. This suggests that the effect of insulin resistance (as a proxy for MetS) would be expected at more elevated levels of BMI. Thus, despite the overall positive relationship between BMI and BMD, more obese individuals experience a relative decrease in BMD in parallel to more severe insulin resistance, a finding which imposes an evaluation of the patient population as a function of degrees of obesity.

### 3.4. Stratification of Population as Lean and OW/OB Patients

We re-evaluated the relative contribution of MetS in OP patients stratified as lean and OW/OB, based on the inflection point (BMI of 27.2 kg/m^2^) as the cutoff value (Table S4). Of note, both groups were of comparable ages. The OW/OB group exhibited 54.3% obesity (BMI ≥ 30 kg/m^2^) and nearly a 2-fold increase in COB obesity compared to lean individuals (Table S4). Metabolic parameters such as glycemia, HOMA-IR, and HbA1c were abnormally elevated in the OW/OB individuals, but without modifications for TG and HDL-C. Insulin resistance was 2.8-fold more prevalent in the OW/OB group, concordant with 50% MetS compared to only 18.9% in lean patients. Among MetS components, the prevalence rates of hyperglycemia and central obesity were significantly different in the OW/OB patients compared to lean individuals. Other parameters remained unchanged, although the OW/OB patients trended toward higher values of CRP, ESR, and fibrinogen. In the OW/OB patients, the actual mean values of BMD at different anatomical sites were increased, but these values were lower than those predicted by BMI. Among bone-related parameters, calcium, (25-OH) vitamin D, PTH, and ALP were non-significantly modified, except for OCN, which was found at lower levels (*p* < 0.007). No significant differences were detected for muscular strength and physical performance, and summary statistics indicated a similar prevalence of abnormal tests.

Next, we analyzed the profiles of OP patients with and without MetS after this new stratification (Table 2). In lean patients, significant differences in MetS were found for BMI, WC, fasting glucose, and HbA1c. HOMA-IR was increased up to 1.9 (*p* < 0.0311), but insulin resistance (nominative) remained non-significant. Among the components of MetS, we noted a higher prevalence of HBP and TG. In logistic regression for MetS, the most influential components in lean individuals were glycemia (*p* < 0.0031, OR 4.14, 95% CI [1.44–11.05]), TG levels (*p* < 0.0126, OR 6.82, 95% CI [1.4–33.12]), and HBP (*p* < 0.0012, OR 11.1, 95% CI [2.14–56.55]). BMD and bone turnover markers were unchanged. A completely different picture was obtained for OW/OB patients with and without MetS. Components for MetS were higher glycemia, HbA1c, and TG levels, and a particularly higher HOMA-IR index up to 3.8. Thus, insulin resistance was present in 65.2% of individuals with MetS compared to only 39.1% in patients without MetS, albeit with significance at *p* < 0.07. As a trend, we also detected an increase in P1NP levels in MetS (*p* < 0.06) from 34.4 to 44.9 ng/mL. In logistic regression, the most influential components for MetS in OW/OB patients were only two factors: HbA1c with *p* < 0.0037, OR 9.6, 95% CI [1.64–55.6] and, with similar power, insulin resistance with *p* < 0.0076, OR 6.7, 95% CI [1.49–30.8]. HOMA-IR was a confounding factor with nominal insulin resistance (*p* < 0.0119, OR 1.8, 95% CI [1.03–3.36]). These data indicate that insulin resistance, per se, appeared as a primary influential factor in more obese OP patients stratified by the BMI corresponding to the BMD inflection point. 

**Figure 2:**
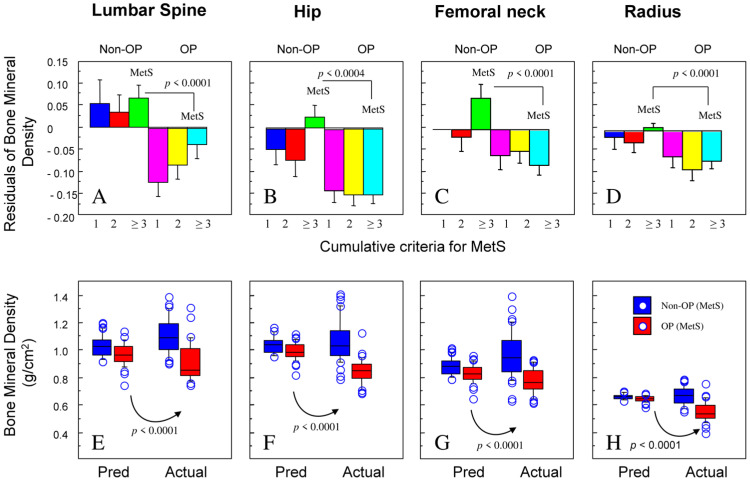
**BMD at different anatomical sites in patients with osteoporosis and MetS compared to predicted values based on BMI.** Predicted values of BMD as function of BMI were calculated in linear regression in subjects without osteoporosis (non-OP). Predicted values were then compared with actual values in non-OP and OP with MetS together (Panels (**E**–**H**)) with the display of residuals (Panels (**A**–**D**)).

To better visualize this challenging situation, we plotted the residuals of BMD from the predicted values based on the BMD–BMI correlation from non-OP subjects. It turned out that the BMD values for MetS were lower than those predicted, and this was observed at all anatomical sites and proportional with the MetS cumulative criteria (Figure 2A–D). Thus, while MetS displayed higher levels of BMD because of the associated obesity, in OW/OB patients, the BMD was relatively lower, indicating a deleterious effect of insulin resistance (Figure 2E–H).

### 3.5. Severe OP with Fractures

The profile of MetS in severe OP with fractures is indicated in Table S5. In severe OP, MetS was at comparable prevalence rates (31% versus 30.6%). Obesity in severe OP was found in 10% versus 26.7% of simple OP cases, but was not statistically different, while the proportion of overweight individuals was higher (28% versus 17% in severe versus simple OP, respectively, *p* < 0.05). 

There were no significant changes in metabolic parameters or bone turnover markers, except for a decreased BMD at the femoral neck, lumbar spine, and hip (but not the radius). Among the muscular and physical performance tests, the TUG test trended to abnormally prolonged values (*p* < 0.053). In the logistic regression for MetS, only glycemia appeared as influential with a high OR of 4.1, 95% CI [1.63–10.39]) and *p* < 0.0005. HbA1c appeared as a trend (*p* < 0.07), albeit with a high OR of 4.9, 95% CI [0.7–31.16]. These data suggest that MetS in severe OP was primarily characterized by high glycemic levels.

## 4. Discussion

In this paper, we characterized MetS and its components in postmenopausal women with OP, concurrently evaluating measured insulin resistance. Through a comprehensive analysis of the correlation between BMD and the cumulative MetS criteria in relation to degrees of obesity, we present evidence for a non-linear, biphasic relationship between BMD and BMI, with a noticeable decline in BMD after a BMI of 27.2 kg/m^2^. Stratifying patients as lean and OW/OB individuals based on the inflection point of BMD revealed a different picture of MetS with strongly associated insulin resistance and high glycemic levels. These data are important for studies on MetS in OP patients, which are often limited to simply reporting the prevalence of MetS. Identifying the deleterious effects of MetS in OP is challenging because, by definition, patients with MetS encompass more individuals with obesity and, consequently, a relatively higher BMD. Our data indicate a relatively lower BMD in OP compared to the predicted values based on the BMIs from non-OP patients.

Although this study did not investigate the mechanistic aspects of insulin resistance, our data suggest that OP, with or without fragility fractures, may be partially explained by a lower BMD. This does not exclude the possibility that defects might also involve a deteriorated bone quality [8]. In the stratified population, the influential factors for MetS were HbA1c as a measure of chronic hyperglycemia and insulin resistance, both with high odds ratios (OR). These findings have significant clinical implications, emphasizing the need to evaluate insulin resistance when investigating MetS in OP.

It is noteworthy that a similar inflection point for BMD was previously observed in a study of healthy Caucasian women aged 18–67 years, corresponding to a proportion of 33% of fat mass [31]. In line with our study, it was noted that, despite a positive correlation between lean mass and BMD, after a threshold of 33% fat mass, a high BMI negatively impacted the BMD at all skeletal sites. While it is challenging to directly compare the value of BMI at the inflection point in this study with our cutoff value of 27.2 kg/m^2^, the authors indicated that a negative impact occurs well before the typical diagnosis of obesity based on a BMI of ≥ 30 kg/m^2^, aligning with our findings. Our observation also aligns with several other studies, although the negative effect of a high BMI varied across different skeletal sites [32,33,34]. More importantly, in a recent study in Taiwanese people, MetS increased the risk for a severe low bone density, and this effect was indeed modulated by BMI [11]. These results are consistent with our observations, although comparing patient stratifications using cutoff values of WC specific for Asian populations remains challenging.

The initial group analysis revealed a MetS prevalence of 30.8%, lower than the 51.5% in non-OP patients, which was explained by a 2.3-fold higher obesity rate in the non-OP group. It is important to note that the prevalence of MetS in OP is not a standalone measure and can be influenced by factors such as the proportion of obese individuals in the studied population. The recruitment of the study population may introduce potential biases, leading to variations in MetS prevalence across different studies. For example, Wong et al. (2016) reported in a review article a MetS prevalence ranging from 10% to 84%, highlighting the variability in these estimates [10]. Muka et al. (2015) reported in European population a MetS prevalence of 45.7% among 1527 women over 55 years in the Rotterdam study [35]. These observed differences in MetS prevalence could be attributed to various factors, including ethnic variations, the use of different MetS definitions, and the potential inclusion of patients with severe OP, who may tend to be leaner. The representation of severe OP patients in the study, particularly those who were only overweight, might have contributed to the lower observed MetS prevalence. For instance, severe OP patients, despite only minor decreases in their mean BMI values, were more frequently overweight (BMI ≥ 25 kg/m^2^). The influence of these factors underscores the complexity and multifaceted nature of MetS in OP populations [36,37,38,39,40].

The characterization of the MetS profile and its components in OW/OB patients revealed the implication of insulin resistance along with high glycemic levels. While the study did not provide direct evidence for the mechanistic role of insulin resistance in bone mineral density (BMD), several hypotheses should be considered. Obesity and elevated insulin levels generally have a positive impact on BMD. However, MetS, as a syndrome associated with insulin resistance, is thought to negatively affect insulin action in the peripheral tissues, including bone [9]. The relationship between insulin resistance (or hyperinsulinemia) and bone health is complex and involves multiple mechanisms. In vitro studies have demonstrated that insulin has an osteoanabolic effect on bone mass [41]. However, the role of hyperinsulinemia in bone metabolism remains debated. High insulin levels, up to a certain point, may reflect insulin resistance, which could have a detrimental effect on BMD. On the other hand, the chronic elevation of insulin, such as that observed in genetic syndromes of severe insulin resistance (Type A syndrome with *Acanthosis Nigricans*, Alstrom syndrome, or Congenital Generalized Lipodystrophy), may positively affect bone through “spillover” mechanisms involving the insulin-like growth factor-1 (IGF-1) receptor Reviewed in Ref. [9]. Besides the potential role of chronic hyperinsulinemia, we cannot exclude the possibility that the relationship between insulin resistance (e.g., MetS) and BMD could be explained at the genetic level.

This relationship between insulin resistance and bone BMD in non-diabetic patients has been studied with varying findings. Some studies suggest a positive association between HOMA-IR or insulin levels and BMD [42,43], while others indicate a negative association with HOMA [44]. Several factors can influence this relationship, including the presence or absence of diabetes (e.g., T2D), the duration of hyperglycemia, the levels of leptin or adiponectin from the adipose tissue, and the impact of high insulin levels on sex hormone-binding globulin (SHBG). Elevated insulin levels may increase sex hormone levels, affecting bone mass [45,46,47,48]. Additionally, these studies acknowledges the limitations of cross-sectional designs in capturing the dynamic relationship between hyperinsulinemia, insulin resistance, and bone health. Hyperinsulinemia may influence bone health before the development of T2D, complicating the understanding of its effects over the lifespan, especially considering factors like chronic hyperglycemia, the accumulation of advanced glycation end products (AGEs), and oxidative stress [49]. There is existing evidence suggesting cross-talk between OCN and insulin resistance in animal models, where OCN expression contributes to the regulation of insulin production and reduction in visceral adiposity [49,50]. However, this study did not observe significant changes in OCN in the context of MetS, except when patients were stratified based on COB. Overall, the intricate interplay between insulin resistance, bone health, and various metabolic factors requires further investigation, especially in longitudinal studies that can capture the dynamic nature of these relationships over time.

The study acknowledges the potential influence of abdominal obesity on the effects of insulin resistance, considering its association with systemic inflammation, increased inflammatory cytokines, and regulatory hormones for bone metabolism [51,52,53]. COB, characterized by the accumulation of abdominal fat, may mediate some of the effects observed in the relationship between insulin resistance and bone health. The data presented in Table 2 indicate that COB was present in all OW/OB individuals with MetS, but the difference compared to non-MetS subjects was not substantial (91.3% vs. 78.6%, respectively). In lean OP patients, COB in MetS was significantly different from that in non-MetS patients (78.6% vs. 43.3%, respectively). When patients were classified by COB, those with abdominal obesity showed a decrease in OCN levels from 22.59 ± 2.23 to 16.9 ± 0.94 ng/mL (*p* < 0.0073, Mann–Whitney). There was also a slight increase in the PTH levels from 38.6 ± 2.6 to 45.6 ± 2.03 pg/mL (*p* < 0.046). However, no significant changes were observed for P1NP or serum beta-crosslaps peptide. Stratifying patients based on COB revealed that MetS occurred in 40.2% of the OP population compared to only 7.6% in lean individuals (*p* < 0.0003, χ^2^). The study suggests that further detailed investigations are necessary to characterize these aspects, considering factors such as changes in body composition associated with aging or the influence of other genetic and environmental factors [54].

In this study, the major determinants of MetS were hyperglycemia, TG levels, and HBP, components that may act independently on bone health. TG may contribute to a reduced risk of fracture, perhaps by interacting with the protein matrix and bone minerals [55]. Arterial hypertension may be associated with a reduced bone mass due to altered urinary calcium excretion [56]. By far, the most studied and perhaps important factor is chronic hyperglycemia (reflected by HbA1c) or fasting glycemia, per se [56]. It is well established that T2D increases the risk of fracture [57,58]. In our study, based on the OR of association, hyperglycemia was a major determinant of MetS. We cannot exclude the possibility that the deleterious effects of insulin resistance on BMD are in fact, driven by high glycemic levels. The effect of chronic hyperglycemia on bone is mediated by the accumulation of bone lesions (microcracks) or an increase in cortical porosity [35]. Of note, in our study, the TBS score was the unique measure of bone architecture, which was indeed decreased in OP compared to non-OP individuals (Table 1), but not significantly changed in MetS (Table 2).

The population of OP patients included in this study was in somewhat heterogenous, since it contained both treated and untreated patients for OP. The proportions of treated and untreated patients with OP were 58.3% and 41.6%, respectively. The fact that the same proportions were found in OW/OB individuals (60,8% and 39.1%, respectively) suggest that there was a poor effect of the treatment on our major finding. Indeed, the introduction of a binary category of treatment (YES/NO) in the logistic regression for MetS showed that the variable was not significant (*p* = 0.2) and the OR of the association of various components varied poorly. For instance, glycemia was associated in all OP patients with an OR of 6.5 95%CI [2.5–16.5] with *p* < 0.0001, and in the presence of treatment, the OR was 7.3 95% CI [2.8–19.3] with the same *p* < 0.0001. In view of the large variability in treatment options in these patients (to limit the fracture risk), much larger studies would be necessary to obtain homogenous treatment subcategories of OP patients.

In regard to this, future studies should also consider variations in different diet regimens. Indeed, the study of OP in postmenopausal women should consider lifestyle factors in the pathogenesis of OP such as smoking status, alcohol consumption, and physical activity, including dietary regimen and potential dietary supplementation to improve the metabolic state. For instance, 6-month dietary surveys of diet indicated that myo-inositol, cocoa polyphenols, and soy isoflavones improved BMI and WC, shifted obese women to overweight, and reduced the prevalence of a diabetic state [59]. In this study, we were unable to estimate these factors in the pathogenesis of MetS in OP, particularly because the study was focused on MetS in relation to insulin resistance and not OP in general. Preliminary results indicate, however, that alcohol consumption and smoking status did not alter the major findings. For instance, in all OP patients, Mets was associated in logistic regression with glycemia, TG, and HPB with ORs of 2.6 (*p* < 0.0001), 3.1 (*p* < 0.0002), and 4.9 (*p* < 0.0001), respectively. After the inclusion of smoking status (YES/NO), the ORs were only very slightly modified (2.5, 3.1, and 5.4, respectively) and the smoking factor itself was non-significant (*p* = 0.28). The impact of alcohol consumption could not be estimated, because none of the subjects reported alcohol use.

If we consider only the OW/OB individuals, similar conclusions were drawn. HBA1c and HOMA were associated with MetS with ORs of 7.5 and 1.8, respectively, while smoking status induced only minor OR changes (8.4 and 1.9, respectively). We cannot exclude the possibility that population size was not sufficient to identify the effects of lifestyle factors. Further studies specifically focused on OP (as a variable) and well controlled are necessary to measure the effect of such additional factors (including dietary surveys).

The non-linear correlation between BMI and BMD warrants a more general discussion concerning strategies and study designs for MetS in OP. The assumption of a protective effect of weight gain in postmenopausal women is longstanding [18,19]. In many publications, MetS has been studied simply by its prevalence or with the BMD adjusted for BMI or age. These approaches have yielded contradictory results [10,11,12,13]. The prominent driving force of BMI for BMD often leads to conclusions about the role of MetS that are, in fact, reflections of obesity. This is especially true when using the IDF definition for MetS, which implies COB as a necessary condition for its diagnosis. The adjustment of BMD is a statistical procedure that does not consider the non-linear correlation between BMD and BMI, potentially masking the detrimental effects of MetS and insulin resistance. In this study, we approached this issue differently by examining the MetS profile and estimating the power of association of its components. After stratifying the patients based on BMI, insulin resistance emerged as an influential factor in MetS in the OW/OB patients, aligning well with the high HOMA-IR values as a function of BMI percentiles (Figure 2D). The prevalence of MetS, unlike the prevalence of a disease (e.g., T2D) in the population, appears to not be very relevant, since the proportion of patients carrying the syndrome essentially depended on that of obesity, subject to recruitment biases. Studying independent components of MetS can provide interesting results. For instance, a recent study on 13,182 free-living Caucasian women in Italy indicated that a HBP, high TG, and low HDL-C increased the risk of a low BMD and OP, while hyperglycemia/T2D decreased this risk. By comparing 7176 patients with low HDL-C and 3702 patients with hyperglycemia and T2D, the overall effect of MetS was pathogenic for OP, albeit with an OR no higher than 1.19 [60]. HOMA-IR was not investigated in this study, but one can speculate that insulin resistance would be proportional to low HDL-C and associated with a decreased BMD and a high risk of OP.

The strength of this study is its ability to demonstrate the deleterious effect of MetS on BMD in correlation with various levels of BMI. More precisely, we identified a biphasic correlation with an inflection point at 27.2 kg/m^2^ of BMI, which corresponds to overweight and obese patients. This observation was possible by dissecting the components of MetS and considering the cumulative criteria for MetS in relation with insulin resistance. Other previous studies, by simply considering the prevalence of MetS in OP, have yielded conflicting results, estimating, in fact, the effect of obesity itself. Such studies concluded a protective effect of MetS on OP in postmenopausal women, which is contradictory to numerous studies on insulin resistance.

We acknowledge that our study has potential limitations. It is possible that our findings could be specific to OP patients recruited in a medical setting, and there may be biases in the recruitment of non-OP patients with a higher BMI compared to OP patients, as well as potential desirability biases. While a more extensive protocol for European populations is ongoing in our laboratory, it should be mentioned that the reported prevalence of MetS is compatible with other reports in Romania. Moreover, the non-linear correlation of BMD with BMI has been observed in other large populations, concurrent with the modulatory effects of BMI on the deleterious effect of MetS. Obviously, the pathogenesis of OP is multifactorial, and further studies in large populations are necessary to investigate how our data fit into a multifactorial context.

Another potential limitation is the use of HOMA-IR as a measure of insulin resistance instead of the gold standards like the euglycemic clamp or intravenous glucose tolerance test (IVGTT). However, the HOMA-IR index has been shown to be a robust parameter in epidemiological studies. We previously established a good correlation between the insulin sensitivity index (Si) in IVGTT, HOMA-IR index, and cumulative criteria for MetS. Limitations of this study also arise from the investigation of biomarkers for inflammation. CRP, fibrinogen, and ESR were useful, but more specific investigations into aspects such as IL-6 and TNF-α may be necessary to better understand the role of insulin resistance in MetS. Similar considerations can be made for the investigation of the bone parameters used for a diminished bone mass and altered bone architecture, which were limited in this study to BMD and TBS.

## 5. Conclusions

By studying the features of MetS in OP, our study highlighted the detrimental impact of MetS components on bone health in a complex interplay with obesity and insulin resistance, revealing a non-linear correlation between BMD and BMI with a specific inflection point that can differently stratify patients for the study of MetS. While standard classification of OP detected some influential factors in MetS, the stratification of patients based on BMI in relation to BMD revealed the pathogenic roles of chronic hyperglycemia and insulin resistance. This research underscores the intricate relationship between OP, MetS, insulin resistance, and obesity in postmenopausal women, emphasizing the need for more elaborate research protocols that take into account the biphasic role of obesity for the design of a more tailored therapeutic approach in postmenopausal OP.

## Figures and Tables

**Table 1:** **Features of non-osteoporotic (non-OP) and osteoporotic (OP) patients.** Subjects were classified according to AACE and severe OP by WHO criteria. Data are presented as mean ± SEM. Groups were compared using Mann–Whitney test (numerical variables) and χ^2^ (nominal variables).

Parameter	Non-OP	OP	*p* ^a^ Value
*N*	68	120	NA
Age (years)	60.7 ± 1.0	66.4 ± 0.7	0.0001
BMI (kg/m^2^)	30.6 ± 0.6	26.2 ± 0.4	0.0001
Waist (cm)	99.5 ± 0.4	92.4 ± 1.1	0.0001
Obesity^BMI^ (%) ^b^	48.5	20.8	0.0001
Fasting glucose (mmol/L)	5.6 ± 0.1	5.4 ± 0.1	NS
Fasting insulin (μU/mL)	9.8 ± 1.2	8.3 ± 0.5	NS
HOMA_IR_^c^	2.6 ± 0.4	2.1 ± 0.2	NS
Insulin resistance (%) ^d^	35.3	32.5	NS
SBP (mm Hg) ^e^	125.8 ± 1.8	122.1 ± 1.6	NS
DBP (mm Hg) ^e^	77.7 ± 1.2	75.8 ± 0.9	NS
HbA1c (%)	5.7 ± 0.1	5.5 ± 0.0	0.0057
TG (mmol/L)	1.2 ± 0.07	1.13 ± 0.0	NS
HDL-cholesterol (mmol/L)	1.54 ± 0.0	1.53 ± 0.0	NS
Central obesity (%)	86.8	67.5	0.02
Hyperglycemia (%)	19.1	18.3	0.032
Hypertension (%)	57.3	40.0	0.027
High TG (%)	19.1	18.3	0.056
Low HDL (%)	44.1	30	NS
MetS^ATPIII^ (%)	51.5	30.8	0.031
Osteopenia (%)	51.5	2.5	0.0001
Severe-OP with fractures (%)	0.0	48.3	NA
BMD lumbar spine (g/cm^2^)	1.1 ± 0.0	0.9 ± 0.0	NA
BMD hip (g/cm^2^)	1.0 ± 0.0	0.8 ± 0.0	NA
BMD femoral neck (g/cm^2^)	0.9 ± 0.0	0.76 ± 0.0	NA
BMD radius 33% (g/cm^2^)	0.7 ± 0.0	0.55 ± 0.0	NA
TBS L1–L4	1.3 ± 0.0	1.2 ± 0.0	0.0001
Osteocalcin (ng/mL)	18.8 ± 0.8	19.0 ± 1.0	NS
P1NP (ng/mL)	47.2 ± 2.4	43.2 ± 2.7	0.02
Beta-crosslaps (ng/mL)	0.4 ± 0.0	0.4± 0.0	NS
SUM^stat^ muscle (%)	22.7	45.0	0.0083

^a^ NS stands for non-significant and NA for non-applicable; ^b^ Obesity was considered based on BMI ≥ 30 kg/m^2^; ^c^ HOMA-IR was calculated as glucose (mmol/L) × Insulin (μU/mL)/22.5; ^d^ insulin resistance was considered as function of HOMA_IR_ values with cut-off 1.92 as mean + 2 SEM of lean non-OP subjects; ^e^ SBP and DBP, stand for systolic and diastolic blood pressure; MetS, Metabolic syndrome; P1NP, Procollagen type I N-terminal propeptide; TBS, trabecular bone score; SUM^stat^, summary statistics of muscular tests, as described under “Patients and methods” section.

**Table 2:** **Features of osteoporosis patients with and without MetS stratified as function of inflection point of BMD.** Patients were stratified as function of inflection point of 27.2 kg/m^2^ for BMI as lean and overweight/obese (OW/OB). Data are presented as mean ± SEM. The two groups non-MetS and MetS were compared using Mann–Whitney test for numerical variable and χ^2^ for nominal variable.

Parameter	Lean BMI 21–27.2 kg/m^2^			Overweight/Obese (OW/OB) BMI 27.2–35.5 kg/m^2^		
	non-MetS	MetS	P ^a^	non-MetS	MetS	P
*n*	60	14	NA	23	23	NA
Age (years)	65.9 ± 1.2	67.0 ± 1.8	NS	64.9 ± 1.5	68.5 ± 0.3	NS
BMI (kg/m^2^)	22.7 ± 0.3	24.5 ± 0.8	0.0067	30.4 ± 0.5	32.2 ± 0.7	NS
Waist (cm)	85.5 ± 1.1	90.4 ± 2.1	0.0353	100.8 ± 1.8	103.1 ± 2.0	NS
Obesity^BMI^ (%) ^b^	0.0	0.0	NA	47.8	60.9	NS
Fasting glucose (mmol/L)	5.0 ± 0.0	5.8 ± 0.2	0.0007	5.3 ± 0.1	6.0 ± 0.2	0.0022
Fasting insulin (μU/mL)	6.3 ± 0.6	7.2 ± 0.9	NS	8.5 ± 0.7	13.7 ± 1.7	0.023
HOMA_IR_^c^	1.4 ± 0.1	1.9 ± 0.3	0.0311	2.0 ± 0.2	3.8 ± 0.5	0.012
Insulin resistance (%) ^d^	18.3	28.6	NS	39.1	65.2	0.07
SBP (mm Hg) ^e^	116.5 ± 2.3	128.8 ± 5.0	0.0241	121.5 ± 2.3	132.6 ± 3.7	0.02
DBP (mm Hg) ^e^	73.0 ± 1.3	79.4 ± 3.0	0.045	75.9 ± 1.7	80.4 ± 2.3	NS
HbA1c (%)	5.4 ± 0.0	5.6 ± 0.0	0.0344	5.5 ± 0.1	5.9 ± 0.1	0.006
TG (mmol/L)	1.0 ± 0.1	1.3 ± 0.2	0.0043	1.0 ± 0.1	1.5 ± 0.1	0.0011
HDL-cholesterol (mmol/L)	1.6 ± 0.0	1.4 ± 0.1	NS	1.5 ± 0.0	1.4 ± 0.1	0.033
Central obesity (%)	43.3	78.6	0.0149	91.3	100.0	NS
Hyperglycemia (%)	15.0	71.4	0.0001	21.7	78.2	0.0001
Hypertension (%)	25.0	71.4	0.0009	26.1	73.9	0.0009
High TG (%)	11.6	28.6	NS	4.3	43.4	0.0025
Low HDL (%)	16.6	42.8	0.0002	17.3	54.5	0.013
Osteopenia (%)	5.0	0.0	NA	0.0	0.0	NA
Severe-OP & fractures (%)	45.0	64.3	NS	56.5	39.1	NS
BMD lumbar spine (g/cm^2^)	0.81 ± 0.1	0.83 ± 0.0	NS	0.89± ± 0.0	0.97 ± 0.0	0.02
BMD hip (g/cm^2^)	0.77 ± 0.0	0.82 ± 0.0	NS	0.87 ± 0.0	0.88 ± 0.0	NS
BMD femoral neck (g/cm^2^)	0.73 ± 0.0	0.76 ± 0.0	NS	0.80 ± 0.0	0.79 ± 0.0	NS
BMD radius 33% (g/cm^2^)	0.52 ± 0.0	0.51 ± 0.0	NS	0.58 ± 0.0	0.59 ± 0.0	NS
TBS L1–L4	1.22 ± 0.0	1.22 ± 0.0	NS	1.25 ± 0.0	1.23 ± 0.0	NS
Osteocalcin (ng/mL)	21.4 ± 1.6	18.8 ± 3.3	NS	14.5 ± 1.3	16.4 ± 1.2	NS
P1NP (ng/mL)	46.1 ± 4.1	42.1 ± 8.4	NS	34.4 ± 4.9	44.9 ± 4.5	0.06
Beta-crosslaps (ng/mL)	0.39 ± 0.0	0.40 ± 0.1	NS	0.31 ± 0.0	0.3 ± 0.0	NS
SUM^stat^ muscle (%)	41.6	50.0	NS	43.5	52.2	NS
Grip strength Right (kg)	20.39 ± 0.7	22.1 ± 1.1	NS	20.4 ± 0.9	20.8 ± 0.8	NS
Grip strength Left (kg)	18.4 ± 0.6	19.5 ± 0.9	NS	19.1 ± 0.9	18.9 ± 0.9	NS
TUG (sec)	12.7 ± 0.9	11.2 ± 1.1	NS	12.2 ± 0.8	12.2 ± 0.8	NS

^a^ NS stands for non-significant and NA for non-applicable; ^b^ Obesity was considered based on BMI ≥ 30 kg/m^2^; ^c^ HOMA-IR was calculated as glucose (mmol/L) × Insulin (μU/mL)/22.5; ^d^ insulin resistance was considered as function of HOMA_IR_ values with cut-off 1.92 as mean + 2 SEM of lean non-OP subjects; ^e^ SBP and DBP, stand for systolic and diastolic blood pressure; MetS, Metabolic syndrome; P1NP, Procollagen type I N-terminal propeptide; TBS, trabecular bone score; SUM^stat^, summary statistics of muscular tests, as described under “material and methods” section.

## Data Availability

Full data may be obtained from the author.

## Supplementary Materials

The following supporting information can be downloaded at: https://www.mdpi.com/article/10.3390/jcm13092529/s1. Table S1: Description of clinical tests for muscular strength and physical performance, Table S2: Additional laboratory features and muscular strength and physical performance in non-OP and OP patients, Table S3: Features of patients with and without MetS among non-OP and OP populations, Table S4: Features of patients with OP stratified as lean and overweight/obese using the cutoff of 27.2 kg/m^2^, Table S5: Features of patients with simple and severe OP with fragility fractures, Figure S1: Correlation between BMD at the 1/3 of radius and cumulative criteria for MetS, Figure S2: Residuals of BMD values at femoral neck and hip anatomical sites as function of the degree of obesity.

## Abbreviations

AACE	American Association of Clinical Endocrinologists
AGEs	Advanced glycation end products
ALP	Alkaline phosphatase
ATP-III	Adult Treatment Panel-III
BMD	Bone mineral density
BMI	Body mass index
COB	Central obesity
CRP	C-reactive protein
CST	Chair stand test
DBP	Diastolic blood pressure
DEXA	Dual X-ray absorptiometry
ECLIA	Electrochemiluminescent immunoassay
ESR	Erythrocyte sedimentation rate
HDL-C	High-density lipoprotein cholesterol
HGS	Handgrip strength
HOMA	Homeostasis Model Assessment
IGF-1	Insulin-like growth factor-1
IOF	International Osteoporosis Foundation
IVGTT	Intravenous glucose tolerance test
LDL-C	Low-density lipoprotein cholesterol
MetS	Metabolic syndrome
NCEP	National Cholesterol Education Program
OB	Obesity
OCN	Osteocalcin
OP	Osteoporosis
OW	Overweight
P1NP	Procollagen type I N-terminal propeptide
PTH	Parathyroid hormone
SBP	Systolic blood pressure
SHBG	Sex hormone-binding globulin
T2D	Type 2 diabetes mellitus
TBS	Trabecular Bone Score
TG	Triglycerides
TUG	Timed up and go test
WC	Waist circumference
WHO	World Health Organisation
