# Supplementary material for: Relative Contribution of Metabolic Syndrome Components in Relation to Obesity and Insulin Resistance in Postmenopausal Osteoporosis
Source: J Clin Med. 2024 Apr 25;13(9):2529. doi: 10.3390/jcm13092529 (PMC11084230; doi:10.3390/jcm13092529)

Journal of
Clinical Medicine

Supplementary Material

Table S1. Description of clinical tests for muscular strength and physical performance. Normal values for each test were obtained from European guidelines EWGSOP-2 [30].

Description	Normal values
**Handgrip strength** (HGS) was measured bilaterally using a calibrated digital handheld dynamometer (KERN MAP 80K1S) with the patient in a sitting position, the shoulder adducted and neutrally rotated, the elbow against the body, flexed at 90 degrees, and the forearm and wrist in a neutral position. Mean data of three trials are expressed kg.	Values ≥ 16 kg (EWGSOP-2)
**Chair Stand Test** (CST) was performed with a standard height chair with a straight back and no armrests and placed next to a wall. Each subject was instructed, at the count of "go," to stand up fully and sit down five times as fast as possible while keeping their arms folded across the chest and without resting on the chair in between repetitions. The patient was not talked during the test to avoid interfering with the patient's speed. The 5xSTS was timed from initial to final seated position and was expressed in seconds.	Values ≤ 15 sec (EWGSOP-2)
**Gait speed (GS)** was performed once and assessed over ground at a fast pace using 4-meter straight walking trajectory with a standing start protocol. Timing[a] was started when the first foot movement was observed and was ceased once the subject had completely crossed the end line. Gait speed was expressed in m/s.	Values > 0.8 m/sec (EWGSOP-2)
**Timed Up and Go test (TUG)** recorded how long it took for a subject to rise from a standard height chair, walk 3 m at a comfortable pace to a mark placed on the floor, turn around, walk back to the starting point, and sit back down on the chair. TUG was expressed in seconds.	Values < 20 sec (EWGSOP-2)
**Tinetti Assessment Tool/Test (TT)** is a physical performance examination that scores specific gait and balance-related tasks on a three-point ordinal scale with a range of 0 to 2, where 0 represents the most impairment and 2 represents independence.	Maximum score is 28 points (12 for gait and 16 for balance). Subjects below 19 and in the range of 19-24 or 25-28 points are at high and medium risk or low fall risk, respectively

a, The time was recorded using a digital stopwatch. The participants wore flat-heeled, comfortable shoes and walking aids were permitted but not required since all participants could walk independently.

Table S2. Additional laboratory features and muscular strength and physical performance in non-OP and OP patients. Data are presented as mean ± SEM. The two groups were compared using Mann-Whitney test for numerical variable and χ^2 for nominal variable. Summary statistics of muscular tests are indicated in Table 1.

**Parameter**	**Non-OP**	**Non-OP**	**P** non-OP/OP
n	68	120	
ESR (mm/h)	20.5 ± 1.4	22.7 ± 3.6	NS
CRP (mg/dl)	0.53 ± 0.1	0.35 ± 0.0	0.039
Fibrinogen (mg/dl)	393.6 ± 8.7	368.5 ± 6.5	0,0221
Uric acid (mg/ml)	4.9 ± 0.2	4.4 ± 0.1	0.0197
Magnesium (mmol/L)	0.089 ± 0.0	0.093 ± 0.0	NS
Calcium (mg/dl)	4.1 ± 0.0	4.1 ± 0.0	NS
Vitamin D (ng/ml)	28.2 ± 1.3	29.8 ± 0.9	NS
PTH (pg/ml)	40.0 ± 1.8	43.4 ± 1.6	NS
ALP (U/L)	66.3 ± 2.4	65.1 ± 1.8	NS
Grip strength Right (kg)	24.2 ± 0.7	20.9 ± 0.4	0.0001
Grip strength Left (kg)	21.8 ± 0.6	18.8 ± 0.4	0.0001
CST (sec)	12.7 ± 0.5	15.5 ± 0.6	0.0015
Gait speed (m/sec)	1.3 ± 0.0	1.1 ± 0.0	0.0001
TUG (sec)	10.1 ± 0.4	12.3 ± 0.5	0.0046
TINETTI test (points)	24.2 ± 0.3	23.2 ± 0.3	0.0163

a, NS stands for non-significant; ESR, erythrocyte sedimentation rate; CRP, C-reactive protein; CST, Chair Stand Test; TUG, Timed Up and Go.

Table S3. Features of patients with and without MetS among non-OP and OP populations. Patients were classified following the AACE definition of osteoporosis in postmenopausal women while severe OP following the WHO criteria. MetS was defined in patients having 3 or more ATP-III criteria. Data are presented as mean ± SEM. The two groups were compared using Mann-Whitney test for numerical variables and χ^2 for nominal variables.

**Parameter**	**Non-OP**			**OP**		
	**non-MetS**	**MetS**	**P[a]**	**non-MetS**	**MetS**	**P**
n	33	35		83	37	
Age (years)	60.3 ± 1.6	61.0 ± 1.3	NS	65.7 ± 0.9	67.9 ± 1.1	NS
BMI (kg/m^2)	27.4 ± 0.7	33.4 ± 0.8	0.0001	24.8 ± 0.5	29.3 ± 0.8	0.0001
Waist (cm)	94.1 ± 1.7	104.4 ± 1.7	0.0001	89.8 ± 1.2	98.3 ± 1.8	0.0001
Obesity^{BMI} (%)	21.2	74.3	0.0001	13.2	37.8	0.0022
Fasting glucose (mmol/L)	5.1 ± 0.1	6.1 ± 0.2	0.0001	5.1 ± 0.1	5.9 ± 0.1	0.0001
Fasting insulin (µU/mL)	6.9 ± 0.8	12.6 ± 2.1	0.0147	6.9 ± 0.5	11.2 ± 1.2	0.0002
$HOMA_{IR}$	1.6 ± 0.2	3.6 ± 0.7	0.0068	1.59 ± 0.1	3.0 ± 0.4	0.0001
Insulin resistance (%)	15.1	54.3	0.0007	24.1	51.3	0.0032
SBP (mmHg)	120.6 ± 1.8	130.6 ± 2.8	0.0047	117.9 ± 1.8	131.1 ± 2.9	0.0001
DBP (mmHg)	76.8 ± 1.7	78.5 ± 1.8	NS	73.8 ± 1.1	80.0 ± 1.8	0.0024
HbA1c (%)	5.5 ± 0.1	5.9 ± 0.1	0.0084	5.4 ± 0.0	5.8 ± 0.0	0.0001
TG (mmol/L)	0.93 ± 0.0	1.5 ± 0.1	0.0001	1.0 ± 0.0	1.4 ± 0.1	0.0001
HDL-cholesterol (mmol/L)	1.7 ± 0.0	1.4 ± 0.1	0.0004	1.6 ± 0.0	1.4 ± 0.0	0.0079
Central obesity (%)	75.7	97.1	0.0093	56.6	91.8	0.0001 (NA)
Hyperglycemia (%)	9.4	68.6	0.0001	16.8	75.7	0.0001 (NA)
Hypertension (%)	34.3	80.0	0.0002	25.3	75.9	0.0001 (NA)
High TG (%)	3.1	31.4	0.0025	9.6	37.8	0.0007 (NA)
Low HDL (%)	9.4	74.3	0.0001	16.8	59.4	0.0001 (NA)
Osteopenia (%)	66.6	37.1	0.03	3.6	0.0	NA
Severe-OP with fractures (%)	NA	NA	NA	48.1	48.6	NS
BMD Lumbar spine (g/cm^2)	1.0 ± 0.0	1.1 ± 0.0	0.0331	0.83 ± 0.0	0.92 ± 0.0	0.0013
BMD Hip (g/cm^2)	0.9 ± 0.0	1.1 ± 0.0	0.0001	0.79 ± 0.0	0.86 ± 0.0	0.0038
BMD Femoral neck (g/cm^2)	0.9 ± 0.0	1.0 ± 0.0	0.0023	0.74 ± 0.0	0.78 ± 0.0	0.0476
BMD Radius 33% (g/cm^2)	0.52 ± 0.0	0.51 ± 0.0	NS	0.58 ± 0.0	0.59 ± 0.0	NS
TBS L1 – L4	1.22 ± 0.0	1.22 ± 0.0	NS	1.25 ± 0.0	1.23 ± 0.0	NS
Osteocalcin (ng/ml)	21.4 ± 1.6	18.8 ± 3.3	NS	14.5 ± 1.3	16.4 ± 1.2	NS
P1NP (ng/ml)	46.1 ± 4.1	42.1 ± 8.4	NS	34.4 ± 4.9	44.9 ± 4.5	0.06
Beta-crosslaps (ng/ml)	0.39 ± 0.0	0.40 ± 0.1	NS	0.31 ± 0.0	0.3 ± 0.0	NS
SUM^{stat} muscle (%)	41.6	50.0	NS	43.5	52.2	NS
Grip strength Right (kg)	20.39 ± 0.7	22.1 ± 1.1	NS	20.4 ± 0.9	20.8 ± 0.8	NS
Grip strength Left (kg)	18.4 ± 0.6	19.5 ± 0.9	NS	19.1 ± 0.9	18.9 ± 0.9	NS
TUG (sec)	12.7 ± 0.9	11.2 ± 1.1	NS	12.2 ± 0.8	12.2 ± 0.8	NS

a, NS stands for non-significant and NA for non-applicable; MetS, Metabolic syndrome; P1NP, Procollagen type I N-terminal propeptide; TBS, trabecular bone score; SUM^{stat} muscle, summary statistics of muscular tests as described in Patients and methods section.

Table S4. Features of patients with OP stratified as lean and overweight/obese using the cutoff of 27.2 kg/m². Data are presented as mean ± SEM. The two groups were compared using Mann-Whitney test numerical variables and χ^2 for nominal variables.

**Parameter**	**Lean** BMI 21-27.2 kg/m^2	**OW / OB** BMI 27.2 -35.5 kg/m^2	**P** **Lean-OW/OB-OP**
n	*74*	*46*	
Age (years)	66.1 ± 1.0	66.7 ± 1.0	NS
BMI (kg/m^2)	23.1 ± 0.3	31.3 ± 0.4	0.0001
Waist (cm)	86.4 ± 1.0	101.9 ± 1.3	0.0001
Obesity (%)	0.0	54.3	0.0001
Fasting glucose (mmol/L)	5.2 ± 0.1	5.7 ± 0.1	0.0017
Fasting insulin (µU/mL)	6.5 ± 0.5	11.2 ± 1.0	0.0001
HbA1c (%)	5.4 ± 0.0	5.7 ± 0.0	0.0014
$HOMA_{IR}$	1.52 ± 0.1	2.9 ± 0.3	0.0001
Insulin resistance (%)	20.3	52.2	0.0002
SBP (mmHg)	118.9 ± 2.1	127.0 ± 2.3	0.0149
DBP (mmHg)	74.3 ± 1.2	78.2 ± 1.5	0.0471
Triglycerides (mmol/L)	1.1 ± 0.0	1.2 ± 0.1	0.07 NS
HDL-cholesterol (mmol/L)	1.6 ± 0.0	1.4 ± 0.4	NS
Central obesity (%)	50.0	95.6	0.0001
Hyperglycemia (%)	74.3	50.0	0.006
Hypertension (%)[c]	66.6	50.0	NS
High Triglycerides (%)	14.9	21.7	NS
Low HDL (%)	21.6	35.5	NS
$MetS^{ATPIII}$ (%)	18.9	50.0	0.0003
CRP (mg/dl)	0.3 ± 0.0	0.4 ± 0.1	NS 0.07
ESR (mm/h)	19.5 ± 1.2	27.7 ± 9.0	NS
Fibrinogen (mg/dl)	358.7 ± 8.3	384.9 ± 10.2	NS
BMD Hip (g/cm^2)	0.8 ± 0.0	0.9 ± 0.0	0.0001
BMD Femoral neck (g/cm^2)	0.7 ± 0.0	0.8 ± 0.0	0.0002
BMD Lumbar spine (g/cm^2)	0.8 ± 0.0	0.9 ± 0.0	0.0001
BMD Radius 1/3 (g/cm^2)	0.5 ± 0.0	0.6 ± 0.0	0.0001
TBS L1 – L4	1.2 ± 0.0	1.2 ± 0.0	NS
Severe OP with Fractures (%)	48.6	47.8	NS
Osteocalcin (ng/ml)	20.8 ± 1.4	15.4 ± 0.9	0.0075
SUM^{stat} muscle (%)	43.2	47.8	NS

Table S5. Features of patients with simple and severe OP with fragility fractures. Data are presented as mean ± SEM. The two groups were compared using Mann-Whitney test numerical variables and χ^2 for nominal variables.

**Parameter**	**Non-OP**	**Non-OP**	**P** non-OP/OP
n	62	58	
Age (years)	65.4 ± 1.0	67.4 ± 1.0	NS
BMI (kg/m^2)	26.6 ± 0.7	25.8 ± 0.6	NS
Waist (cm)	92.8 ± 1.4	91.8 ± 1.6	NS
Obesity (%)	26.7	10.0	NS
Overweight (%)	17	28	0.05
OW/OB (%)	38.7	37.9	NS
Fasting glucose (mmol/L)	5.4 ± 0.1	5.3 ± 0.1	NS
Fasting insulin (µU/mL)	8.9 ± 0.9	7.7 ± 0.7	NS
HbA1c	5.5 ± 0.0	5.5 ± 0.0	NS
$HOMA_{IR}$	2.3 ± 0.3	1.8 ± 0.2	NS
Insulin resistance (%)	30.6	34.4	NS
SBP (mmHg)	119.4 ± 2.4	125.1 ± 2.1	NS
DBP (mmHg)	74.9 ± 1.4	76.7 ± 1.2	NS
Triglycerides (mmol/L)	1.2 ± 0.1	1.0 ± 0.0	NS
HDL-cholesterol (mmol/L)	1.5 ± 0.0	1.6 ± 0.0	NS
Central obesity (%)	65.3	70.0	NS
Hyperglycemia (%)	36.0	27.5	NS
Hypertension (%)	32.0	52.7	NS
High Triglycerides (%)	21.3	15.0	NS
Low HDL-C (%)	29.3	30.0	NS
$MetS^{ATPIII}$ (%)	30.6	31.0	NS
CRP (mg/dl)	0.37 ± 0.1	0.3 ± 0.0	NS
ESR (mm/h)	26.3 ± 6.9	18.9 ± 1.5	NS
Fibrinogen (mg/dl)	372.3 ± 9.6	364.3 ± 8.7	NS
BMD Hip (g/cm^2)	0.84 ± 0.0	0.79 ± 0.0	0.0164
BMD Femoral neck (g/cm^2)	0.78 ± 0.0	0.73 ± 0.0	0.0029
BMD Lumbar spine (g/cm^2)	0.88 ± 0.0	0.83 ± 0.0	0.058
BMD Radius 1/3 (g/cm^2)	0.55 ± 0.0	0.54 ± 0.0	NS
TBS L1 – L4	1.2 ± 0.0	1.2 ± 0.0	NS
Calcium (mg/dl)	4.1 ± 0.0	4.1 ± 0.0	NS
(25-OH) Vitamin D (ng/ml)	29.1 ± 1.4	30.6 ± 1.2	NS
PTH (pg/ml)	42.6 ± 2.4	44.2 ± 2.2	NS
ALP (U/L)	63.1 ± 2.3	67.2 ± 2.9	NS
Osteocalcin (ng/ml)	17.7 ± 1.1	20.0 ± 1.7	NS
P1NP (ng/ml)	39.1 ± 3.0	47.5 ± 4.3	NS
Beta-crosslaps (ng/ml)	0.33 ± 0.0	0.41 ± 0.0	NS
Grip strength Right (kg)	21.6 ± 0.5	20.3 ± 0.6	NS
Grip strength Left (kg)	19.4 ± 0.5	18.0 ± 0.5	NS
CST (sec)	14.7 ± 0.7	16.3 ± 0.9	NS
Gait speed (m/sec)	1.1 ± 0.0	1.0 ± 0.0	NS
TUG (sec)	11.3 ± 0.5	13.4 ± 0.9	0.0530
TINETTI test (points)	23.5 ± 0.3	22.9 ± 0.4	NS
Cumulative abnormal muscle tests (%)			
1	61.3	42.5	NS
2	26.7	40.0	NS
3	9.3	10.0	NS
4	1.3	7.5	NS
SUM^{stat} muscle	37.3	57.5	NS
Beta-crosslaps (ng/ml)	0.33 ± 0.0	0.41 ± 0.0	NS
Grip strength Right (kg)	21.6 ± 0.5	20.3 ± 0.6	NS
Grip strength Left (kg)	19.4 ± 0.5	18.0 ± 0.5	NS
CST (sec)	14.7 ± 0.7	16.3 ± 0.9	NS

Fig S1. **Correlation between BMD at the 1/3 of radius and cumulative criteria for MetS.**

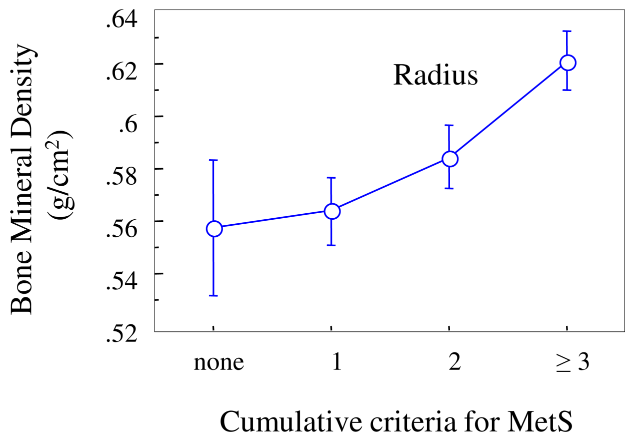

Fig. S2 Residuals of BMD values at femoral neck and hip anatomical sites as function of the degree of obesity. Categories A to F correspond to centiles of BMI in the entire populations (non-OP and OP). Residuals were obtained by subtraction of predicted values of BMD based on regression in non-OP patients from genuine values. Bars indicate the SEM.

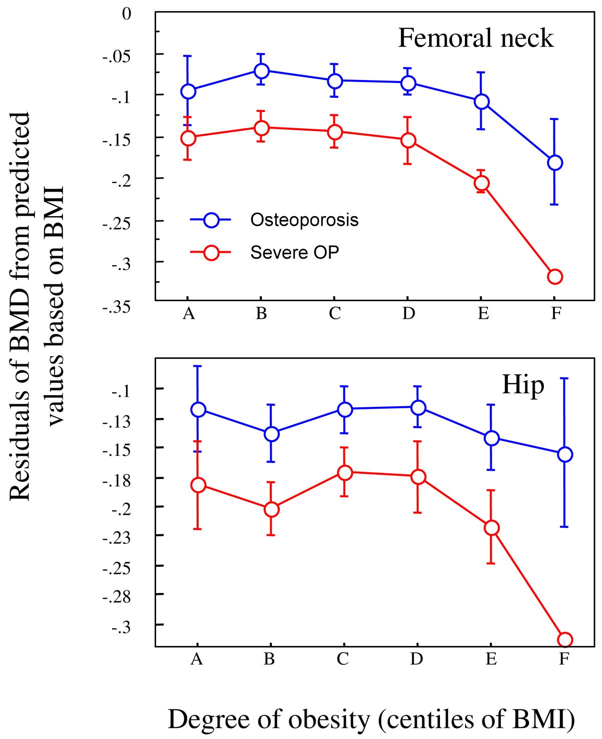

Supplement: Supplementary file 1 [file jcm-13-02529-s001.zip › jcm-2931466-supplementary.pdf]
